# Myoferlin controls mitochondrial structure and activity in pancreatic ductal adenocarcinoma, and affects tumor aggressiveness

**DOI:** 10.1038/s41388-018-0287-z

**Published:** 2018-05-03

**Authors:** Gilles Rademaker, Vincent Hennequière, Laura Brohée, Marie-Julie Nokin, Pierre Lovinfosse, Florence Durieux, Stéphanie Gofflot, Justine Bellier, Brunella Costanza, Michael Herfs, Raphael Peiffer, Lucien Bettendorff, Christophe Deroanne, Marc Thiry, Philippe Delvenne, Roland Hustinx, Akeila Bellahcène, Vincent Castronovo, Olivier Peulen

**Affiliations:** 1 Metastasis Research Laboratory, GIGA Cancer, University of Liège, Liège, Belgium; 2 Laboratory of Connective Tissue Biology, GIGA Cancer, University of Liège, Liège, Belgium; 3 Department of Nuclear Medicine and Oncologic Imaging, University Hospital Center, University of Liège, Liège, Belgium; 4 Tissue Biobank, University Hospital, University of Liège, Liège, Belgium; 5 Laboratory of Experimental Pathology, GIGA Cancer, University of Liège, Liège, Belgium; 6 Laboratory of Neurophysiology, GIGA Neurosciences, University of Liège, Liège, Belgium; 7 Laboratory of Cellular and Tissular Biology, GIGA Neurosciences, University of Liège, Liège, Belgium

## Abstract

Pancreatic ductal adenocarcinoma (PDAC) is the third leading cause of cancer-related death. Therapeutic options remain very limited and are based on classical chemotherapies. Energy metabolism reprogramming appears as an emerging hallmark of cancer and is considered a therapeutic target with considerable potential. Myoferlin, a ferlin family member protein overexpressed in PDAC, is involved in plasma membrane biology and has a tumor-promoting function. In the continuity of our previous studies, we investigated the role of myoferlin in the context of energy metabolism in PDAC. We used selected PDAC tumor samples and PDAC cell lines together with small interfering RNA technology to study the role of myoferlin in energetic metabolism. In PDAC patients, we showed that myoferlin expression is negatively correlated with overall survival and with glycolytic activity evaluated by ^18^F-deoxyglucose positron emission tomography. We found out that myoferlin is more abundant in lipogenic pancreatic cancer cell lines and is required to maintain a branched mitochondrial structure and a high oxidative phosphorylation activity. The observed mitochondrial fission induced by myoferlin depletion led to a decrease of cell proliferation, ATP production, and autophagy induction, thus indicating an essential role of myoferlin for PDAC cell fitness. The metabolic phenotype switch generated by myoferlin silencing could open up a new perspective in the development of therapeutic strategies, especially in the context of energy metabolism.

## Introduction

Pancreatic ductal adenocarcinoma (PDAC) is one of the deadliest forms of cancer [1], usually clinically silent at early stage, it is most frequently diagnosed at an advanced stage. This late diagnosis contributes to one of the lowest 5-year survival rate (<5%). Therapeutic options remain very limited even if some progress were achieved in the development of combination therapies. However, these are still based on classical chemotherapies that are difficult to tolerate and increase only modestly the survival [2]. Unfortunately, targeted therapies able to decrease harmful side effects were unsuccessful in this disease [3]. Consequently, new therapeutic strategies and targets are required to develop effective treatment.

Cells predominantly use glucose to generate ATP through glycolysis with production of lactate, or through glycolysis followed by pyruvate metabolism in Krebs cycle, and oxidative phosphorylation (OXPHOS) in mitochondria [4]. Cells frequently use both pathways, although one of them predominates. In cancer patients, ^18^F-deoxyglucose positron emission tomography (^18^F-DG-PET) is used as a diagnostic and staging tool. In pancreas cancer, ^18^F-DG-PET allows the imaging of tumor glucose metabolism by reflecting glucose transporter and hexokinase-2 expression [5]. The standardized uptake value (SUV) is a semi-quantitative estimation of the ^18^F-DG distribution in the tissues, reflecting glucose metabolism. The metabolic tumor volume (MTV) expressed the metabolically active volume of the tumor while the total lesion glycolysis (TLG) combines SUV and MTV information [6].

Energy metabolism reprogramming, an emerging hallmark of cancer, is necessary for tumor initiation and progression [7]. In PDAC, targeting the ways cancer cells uptake and use nutrients has been considered a therapeutic approach with considerable potential [8]. Several studies reveal an extended metabolic heterogeneity among pancreatic cancer cells [9–13]. Remarkably, only a subset of PDAC cell lines is sensitive to a glucose metabolism modulator (GNE-140) while the other cells compensate owing to an increased OXPHOS [13]. Similarly, mutated KRAS ablation in a PDAC mouse model leads to tumor shrinkage. However, a cell fraction survives and is responsible for tumor relapse. These cells rely on OXPHOS for survival [9]. Consequently, specific OXPHOS inhibitors were developed, showing a selective efficacy in PDAC cell lines and patient-derived xenografts [14, 15].

The first step initiating metabolism is the nutrient uptake through specific transporters. The abundance of these proteins at the plasma membrane is controlled by several steps including exocytosis, endocytosis, and recycling. Myoferlin is a 230 kDa, multiple C2-domain, ferlin family member protein, mainly known for its function in cell fusion as well as endocytosis in myoblasts [16, 17] and endothelial cells [18]. Previously, we described the overexpression of myoferlin in PDAC [19] and its involvement in cancer cell plasma membrane biology such as exocytosis, endocytosis, and recycling [20–22]. In PDAC, myoferlin has a tumor-promoting function by increasing cell proliferation [20]. Recently, we reported myoferlin as a regulator of lipid metabolism in triple-negative breast cancer cells [23]. However, its mechanism of action remains poorly understood.

In the continuity of our previous studies aiming at understanding the role of myoferlin in cancer, we have sought to investigate myoferlin in the context of energy metabolism in PDAC. We found out that myoferlin expression is negatively correlated with tumor size and glycolytic activity evaluated by ^18^F-DG-PET, and overall patient survival. We also showed that myoferlin is more abundant in lipogenic pancreatic cancer cell lines than in glycolytic one, and is required to maintain a high OXPHOS activity. Consistently, myoferlin silencing induced a disorganized mitochondrial network suggesting a mitochondrial fission leading to a decrease of cell proliferation, ATP production, and autophagy induction in PDAC cells.

## Results

### In PDAC patients, myoferlin expression negatively correlates with overall survival and ^18^F-deoxyglucose PET scan data

**Fig. 1:**
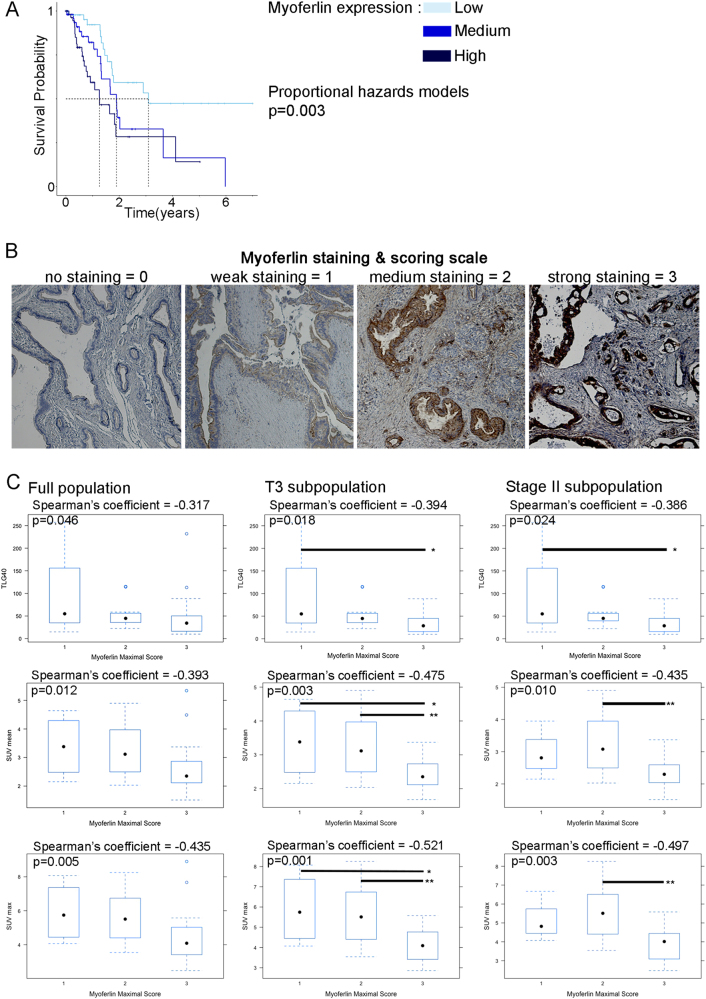
Correlation between myoferlin expression, survival and ^18^F-deoxyglucose PET scan data in PDAC patients. **a** TCGA-PAAD data (http://cancergenome.nih.gov/) were analyzed for overall survival according to their myoferlin gene expression split in tertiles (low expression *N* = 59, medium expression *N* = 59, high expression *N* = 61). Kaplan–Meier curve was established and log-rank probability calculated. **b** FFPE PDAC sections were obtained from institutional tissue biobank and stained for myoferlin. Scoring was performed by three independent investigators. Staining scores were defined as 0 for no staining, 1 for weak staining, 2 for medium staining, and 3 for strong staining. **c** Forty PDAC sections were stained and scored for myoferlin. Spearman’s rank correlation coefficients were calculated between ^18^F-DG PET data (total lesion glycolysis—TLG40, standardized uptake values—SUVmean and SUVmax) and myoferlin scores in total population and T3 or stage II sub-population. Each data point represents median with interquartile range. **P < 0.01 and *P 0.05

Taking advantage of The Cancer Genome Atlas (TCGA) data for a pancreatic cancer cohort (*n* = 179), we performed an overall survival analysis in accordance with the expression level of the myoferlin gene (Fig. 1a). Kaplan–Meier analysis revealed that myoferlin expression was significantly associated with the survival. Patients with a high myoferlin expression have a significantly shorter survival than patients with low myoferlin expression (*P* = 0.0026). The median survival times were, respectively, 460 days, 691 days, and 1130 days for high, medium, and low myoferlin-expressing patients. We have recently shown the same correlation in breast cancer patients [23]. Owing to this previous work, we sought to evaluate the myoferlin abundance in PDAC tumors with different metabolic activity. A cohort of 40 PDAC cases (Supplemental Table S1) with available ^18^F-DG-PET data were stained and scored for myoferlin (Fig. 1b). Myoferlin scores were correlated with PET scan indices (Fig. 1c). It appeared first that myoferlin score was negatively correlated with the tumor size (*ρ* = −0.371, *P* = 0.019). The significance of the correlation increased when T3 (*ρ* = −0.488, *P* = 0.003) or stage II (*ρ* = −0.484, *P* = 0.004) sub-population was selected. Total lesion glycolysis (TLG40) and standardized uptake value (SUVmean, SUVmax) were negatively correlated with the myoferlin maximal score of the tumor, indicating that the more a PDAC is glycolytic, the less it has myoferlin. However, none of these PET indices was correlated with overall survival (TLG40 *P* = 0.16; SUVmean *P* = 0.22; SUVmax *P* = 0.22).

### Myoferlin is highly expressed in lipogenic pancreatic cell lines

**Fig. 2:**
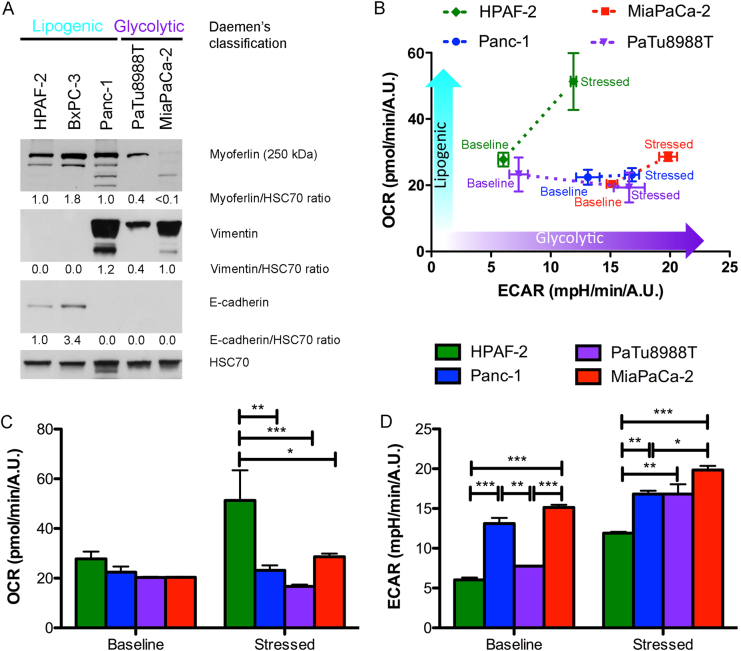
Characterization of PDAC cell lines. HPAF-2, BxPC-3, Panc-1, PaTu8988T, and MiaPaCa-2 cell lines were characterized for **a** myoferlin, vimentin, and E-cadherin expression. HSC70 was used as a loading control. **b** Combined representation of oxygen consumption rate (OCR) and extracellular acidification rate (ECAR) before (baseline) and after (stressed) oligomycin (1 µM) and FCCP (1 µM) addition. **c** OCR and **d** ECAR are represented individually before (baseline) and after (stressed) oligomycin and FCCP addition. Each data point represents mean ± SD, *n* = 3. ***P < 0.001, **P < 0.01, and *P < 0.05

Stricken by these unforeseen correlations in patients, we decided to investigate the link between myoferlin expression and metabolism status of PDAC cell lines. Daemen et al. [11] have recently classified PDAC cell lines into two distinct metabolic subtypes: glycolytic and lipogenic, the latter largely depending upon OXPHOS. These metabolic subtypes correlated significantly with the original Collisson’s classification [24]. The glycolytic subtype corresponds to a mesenchymal phenotype when most of the lipogenic subtype corresponds to an epithelial one. We first evaluated the myoferlin abundance in five PDAC cell lines belonging to the two subtypes described by Daemen et al. [11]. Interestingly, we observed a >2.5-fold higher expression of myoferlin in the lipogenic cell lines, HPAF-2, BxPC-3, and Panc-1, than in the glycolytic PaTu8988T and MiaPaCa-2 cell lines (Fig. 2a). HPAF-2 and BxPC-3 expressed E-cadherin suggesting an epithelial phenotype, while Panc-1, PaTu8988T, and MiaPaCa-2 strongly expressed the vimentin in agreement with their mesenchymal phenotype. In order to validate our cell lines at the light of Daemen’s subtypes, we performed an energetic phenotyping of four selected cell lines: the highly lipogenic HPAF-2, the highly glycolytic MiaPaCa-2, and the intermediary Panc-1 and PaTu8988T cell lines. BxPC-3 cell line was excluded due to its discrepancy with the Collisson’s mesenchymal classification and to its wild-type KRAS status, corresponding to <5% of PDAC cases. In baseline condition, combination of oxygen consumption rate (OCR) and extracellular acidification rate (ECAR) revealed that HPAF-2 cell line was the less glycolytic and the most oxidative when MiaPaCa-2 cell line was the more glycolytic and the less oxidative. Under energetically stressed conditions, the same observation was made but Panc-1 and PaTu8988T cell lines became slightly less oxidative than MiaPaCa-2 cell line (Fig. 2b–d).

### Myoferlin is required to maintain high OXPHOS activity in lipogenic cell lines

**Fig. 3:**
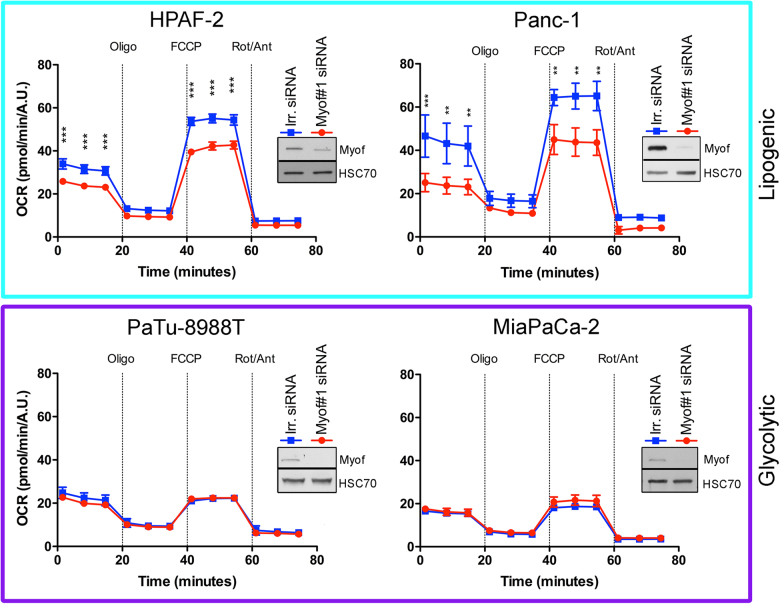
Oxygen consumption rate (OCR) in PDAC cell lines after myoferlin depletion. Kinetic oxygen consumption rate response of HPAF-2, Panc-1, PaTu8988T, and MiaPaCa-2 cells to oligomycin (oligo, 1 µM), FCCP (1.0 µM, except 0.5 µM for MiaPaCa-2), rotenone, and antimycin A mix (Rot/Ant, 0.5 µM each). Upon assay completion, cells were methanol/acetone fixed, and cell number was evaluated using Hoechst incorporation (arbitrary unit, A.U.). One representative experiment out of three is illustrated here. Same results were obtained with the second myoferlin siRNA. Each data point represents mean ± SD, *n* = 3. ***P < 0.001, **P < 0.01. Western blot inserts represented the myoferlin silencing validation

We next inhibited myoferlin translation using small interfering RNA (siRNA) and monitored the OCR after successive addition of oligomycin, FCCP, and rotenone/antimycin A mix. Globally, OCR was higher in lipogenic cell lines than in glycolytic ones (Fig. 3). Myoferlin silencing significantly reduced the OCR in basal condition and after FCCP addition in HPAF-2 and Panc-1 lipogenic cell lines, while no significant decrease was observed in the glycolytic PaTu8988T and MiaPaCa-2 cell lines (Fig. 3). Analysis of respiration compartments (Supplemental Figure S1) revealed that basal, ATP production-related, maximal, and non-mitochondrial OCR were significantly decreased upon myoferlin silencing in both lipogenic cell lines. Spare capacity and proton leakage OCR were also reduced in the highly oxidative HPAF-2 cell line. These results suggest that myoferlin is needed to maintain a high and efficient OXPHOS activity in lipogenic cell lines.

### Myoferlin depletion induces an energetic metabolic switch in lipogenic cell lines

**Fig. 4:**
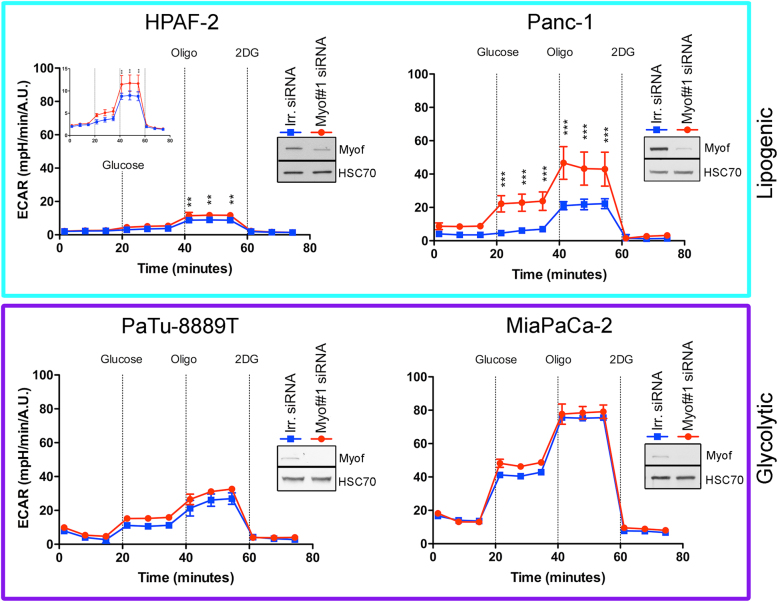
Extracellular acidification rate (ECAR) in PDAC cell lines after myoferlin silencing. Kinetic oxygen consumption rate response of HPAF-2 (insert represents the same data with a specific scale), Panc-1, PaTu8988T, and MiaPaCa-2 cells to glucose (10 mM), oligomycin (oligo, 1 µM), and 2-deoxyglucose (2DG, 50 mM). Upon assay completion, cells were methanol/acetone fixed, and cell number was evaluated using Hoechst incorporation (arbitrary unit, A.U.). One representative experiment out of three is illustrated here. Same results were obtained with the second myoferlin siRNA. Each data point represents mean ± SD, *n* = 3. ***P < 0.001, **P < 0.01. Western blot inserts represented the myoferlin silencing validation

Considering our findings, we then tested whether myoferlin depletion was able to induce an energetic metabolic switch by promoting glycolysis. In order to examine the effect of myoferlin silencing on glycolysis, we analyzed ECAR after successive addition of glucose, oligomycin, and 2-deoxyglucose (Fig. 4). Globally, ECAR was higher in the glycolytic PaTu8988T and MiaPaCa-2 cell lines in comparison with the lipogenic cell lines. Myoferlin depletion induced a significant increase of ECAR in lipogenic cells, while no modification was observed in the glycolytic ones. In HPAF-2 cells, myoferlin silencing increased ECAR after oligomycin addition. However, in Panc-1 cells, myoferlin depletion increased ECAR after glucose and oligomycin addition. Investigating the glycolysis compartments (Supplemental Figure S2) revealed that the glycolytic ECAR was significantly increased in both lipogenic cell lines when the maximal ECAR capacity was significantly increased only in Panc-1 cells. Our results show that the myoferlin-depleted lipogenic cells tried to compensate the OXPHOS deficiency by increasing the glycolysis.

### Myoferlin is required to maintain mitochondrial network structure

**Fig. 5:**
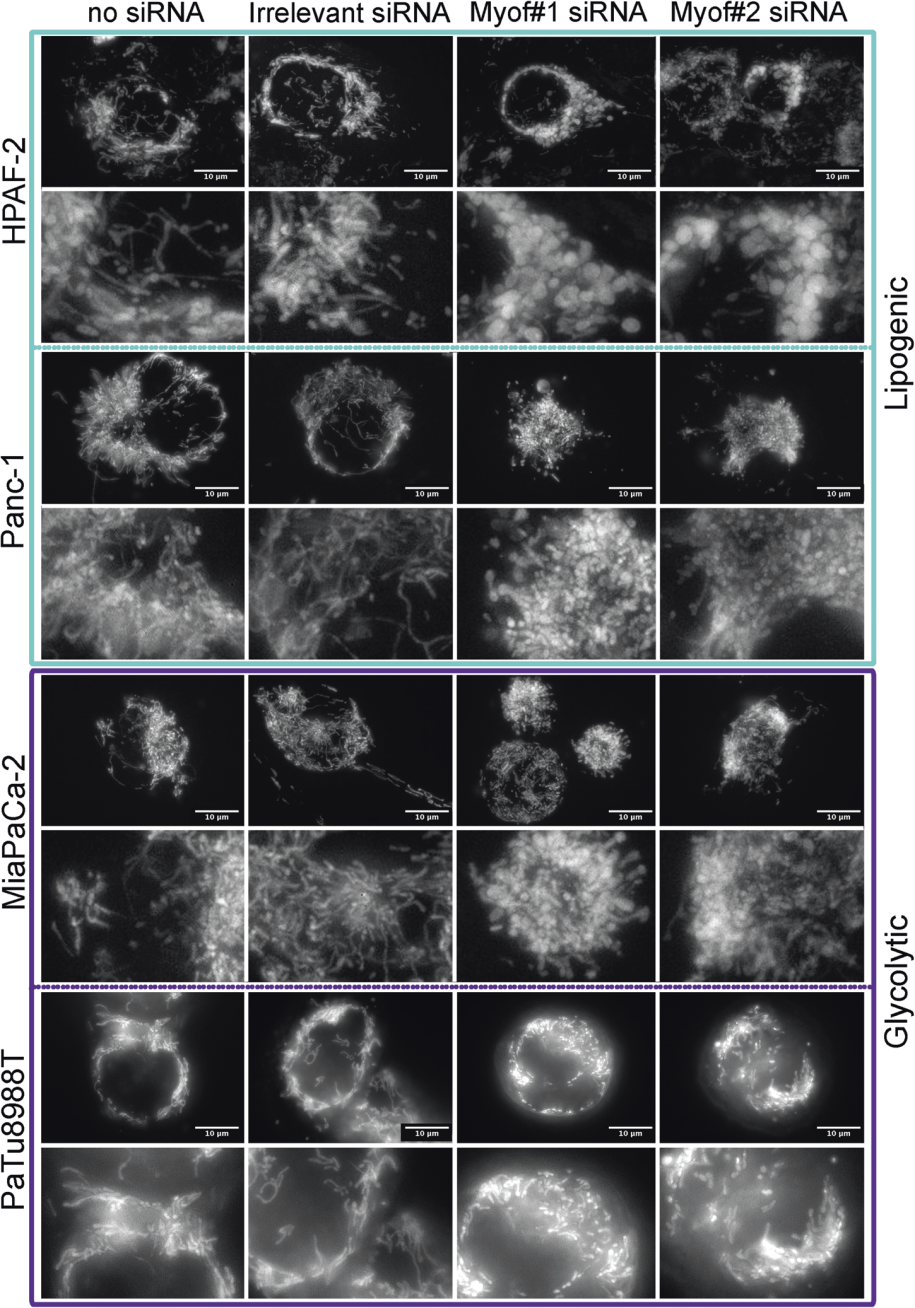
Mitochondrial network visualization after myoferlin silencing. Tetramethyl rhodamine ethyl ester (TMRE) was used to stain mitochondria in living cells. At 48 h post transfection, cells were seeded in μ-Slides 8-well at low confluence then loaded with TMRE (1 nM). Representative experiment out of three

Intrigued by the result we obtained, we decided to evaluate the global mitochondrial morphology (Fig. 5). In HPAF-2 cell line, under control conditions (no siRNA and irrelevant siRNA), tetramethyl rhodamine ethyl ester (TMRE)-stained mitochondria appeared to be a dense network surrounding the nucleus. Myoferlin silencing led to a disruption of this network and to a swelling of the mitochondria. Panc-1 cell line revealed an abundant mitochondrial network. In this cell line, myoferlin depletion led to a complete disorganization of the network and mitochondria appeared to be independent dots. In glycolytic cell lines (MiaPaCa-2 and PaTu8988T), surprisingly, the mitochondrial network was as abundant as in the previous lipogenic cell lines, and myoferlin silencing induced the same morphological modifications than in the lipogenic cell lines. These observations were confirmed by immunofluorescence, using a mitochondrial-specific antibody (Supplemental Figure S3). Taken together, these results suggest for the first time that myoferlin depletion induces mitochondrial fission in PDAC cell lines independently of their energetic metabolism. Myoferlin-depleted Panc-1 cells were sent to transmission electron microscopy in order to assess the mitochondrial ultrastructure (Supplemental Figure S4). In untransfected cells, or transfected with irrelevant siRNA, mitochondria appeared as circular or elongated structures with well-defined cristae, indicating a structural “orthodox state.” In myoferlin-depleted cells, most of the intact mitochondria appeared as circular structures supporting the fission of the mitochondrial network described. Moreover, careful examination of mitochondria indicated a mitochondrial matrix condensation in the myoferlin-depleted conditions, in agreement with a structural “condensed state” and a decreased activity [25].

### Myoferlin depletion increases DRP-1 abundance and induces its phosphorylation

**Fig. 6:**
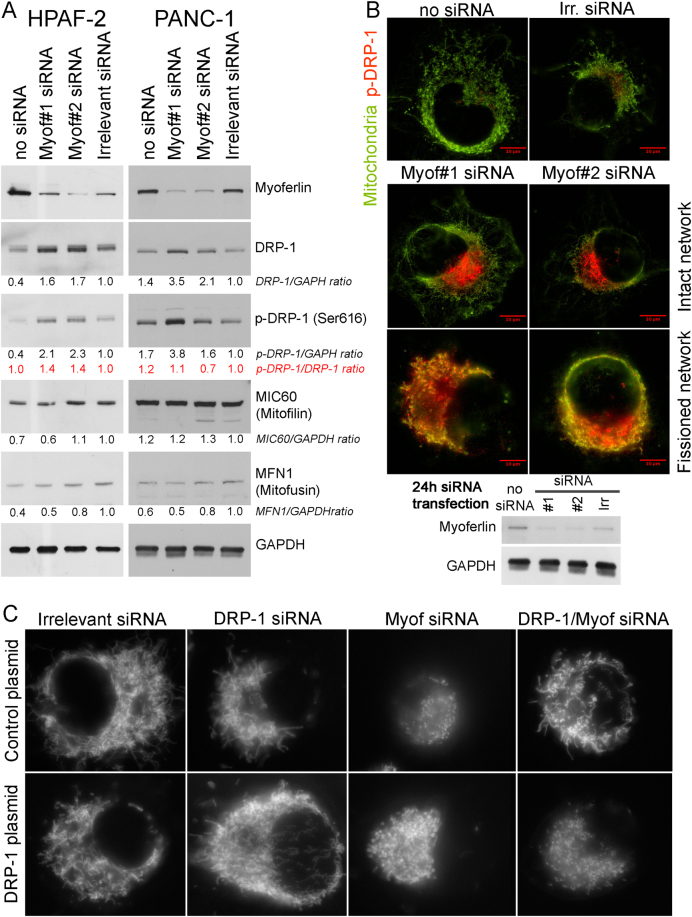
Immunodetection of DRP-1, phospho-DRP-1, mitofilin, or mitofusin in myoferlin-silenced in PDAC cells. **a** Total protein extract (10 µg) from HPAF-2 or PANC-1 cells were subjected to SDS-PAGE followed by western blot analysis with specific antibodies against myoferlin, DRP-1, phospho-DRP-1 (ser616), MIC60/mitofilin, or Mitofusin-1. GAPDH was used as a loading control. **b** Colocalization of phospho-DRP-1 (ser616) and mitochondrial 60 kDa nonglycosylated protein in Panc-1 cells 24 h after myoferlin silencing. Total protein extract (10 µg) were subjected to SDS-PAGE followed by western blot analysis with specific antibodies against myoferlin. HSC70 was used as a loading control. **c** Tetramethyl rhodamine ethyl ester (TMRE) was used to stain mitochondria in living cells. At 48 h post siRNA transfection, cells expressing or not exogenous DRP-1 were seeded in μ-Slides 8-well at low confluence then loaded with TMRE (1 nM). Representative experiments out of three

In order to confirm the mitochondrial fission, we investigated the abundance of several proteins involved in mitochondrial morphology maintenance in HPAF-2 and PANC-1. Dynamin-related protein (DRP)-1 is mainly implicated in mitochondrial fission [26]. Our results revealed a twofold increase in DRP-1 abundance after myoferlin silencing (Fig. 6a). The same increase was observed concerning the phospho-DRP-1 (Ser616) known to be a fission activator [27]. However, abundance of mitofilin—that is mainly involved in controlling cristae morphology [28]—and mitofusin—that mediates mitochondrial clustering and fusion [29]—were not altered by myoferlin depletion (Fig. 6a). In Panc-1 cells, using immunofluorescence, we localized phospho-DRP-1 (Ser616) on mitochondria (Fig. 6b). The experimental time-point was set at 24 h instead of 48 h after siRNA transfection in order to allow the observation of cells with intact mitochondrial network. In control cells (no siRNA or irrelevant siRNA), the mitochondrial network appeared intact with a low DRP-1 staining at one nuclear pole. In myoferlin-silenced cells the DRP-1 staining increased dramatically. In myoferlin-silenced cells that still have an intact mitochondrial network, the DRP-1 staining is also located at a nuclear pole, whereas, in myoferlin-silenced cells that had already undergone the mitochondrial fission, the DRP-1 staining was observed with a full colocalization with the mitochondrial staining. This observation confirms the role of myoferlin in the maintenance of an intact mitochondrial network. In order to demonstrate the role of DRP-1 in the mitochondrial fission induced by myoferlin depletion, we cotransfected Panc-1 cells with siRNA against myoferlin and DRP-1 (Supplemental Figure S5). Immunofluorescence showed that mitochondrial network was kept intact when myoferlin and DRP-1 were depleted at the same time. In order to ultimately prove that the mitochondrial fission induced by myoferlin depletion is indeed mediated by DRP-1, we decided to rescue the mitochondrial fission by an exogenous DRP-1 expression after the cosilencing of myoferlin and endogenous DRP-1. We showed that exogenous DRP-1 was able to induce the mitochondrial fission when both myoferlin and endogenous DRP-1 were inactivated (Fig. 6c). This observation indicates that the mitochondrial fission induced by the myoferlin depletion occurs by the recruitment of DRP-1.

### Myoferlin-induced mitochondrial fission reduces energy production and triggers autophagy

**Fig. 7:**
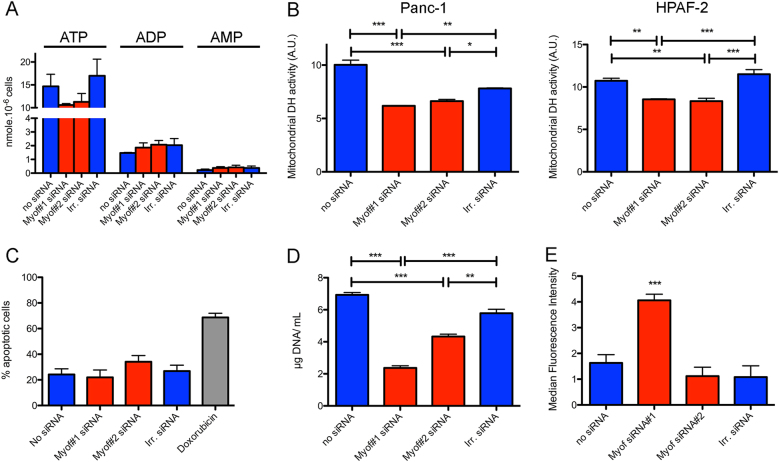
Effects of myoferlin silencing on cell physiology. **a** ATP, ADP, and AMP quantity (nmol) in 10^6^ Panc-1 cells. **b** Succinate-tetrazolium dehydrogenase activity (WST-1 assay). Upon assay completion, cells were methanol/acetone fixed and cell number was evaluated using Hoechst incorporation (arbitrary unit, A.U.). **c** Apoptotic Panc-1 percentage measured by annexin V/PI flow cytometry. Doxorubicin was used as an apoptosis-inducing positive control. **d** Panc-1 cell growth assayed by Hoechst incorporation. **e** ROS accumulation in Panc-1 cells after myoferlin silencing. At 48 h after myoferlin silencing, cells were harvested and loaded with CM-H2DCFDA (2 µM) for 15 min at 37 °C. Then, fluorescence was measured by flow cytometry and analyzed as a median fluorescence intensity. Each data point represents mean ± SD, *n* = 3. ***P < 0.001, **P < 0.01, and *P < 0.05

**Fig. 8:**
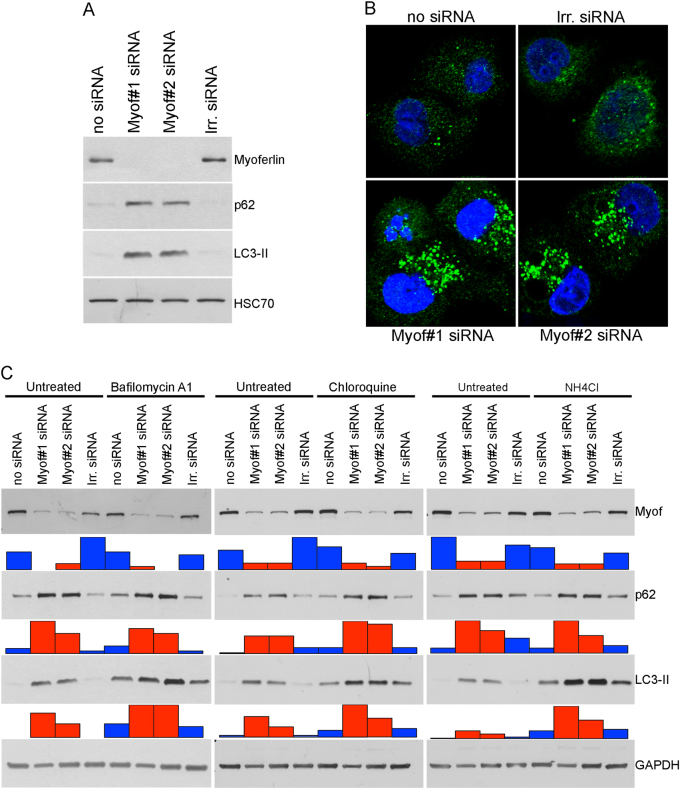
Effects of myoferlin silencing on autophagy. **a** Panc-1 total protein extract (10 µg) were subjected to SDS-PAGE followed by western blot analysis with specific antibodies against myoferlin, p62, and LC3-II. HSC70 was used as a loading control. **b** Immunodetection of LC3-II puncta (autophagosomes) in fixed and permeabilized Panc-1 cells. **c** Autophagic flux analysis 48 h after myoferlin silencing, Panc-1 cells were treated with bafilomycin A1 (200 nM), chloroquine (50 µM), NH4Cl (20 mM), or their respective vehicles for 2 h. Total protein extract (20 µg) were subjected to SDS-PAGE followed by western blot analysis with specific antibodies against myoferlin, p62, and LC3-II. GAPDH was used as a loading control. Histograms below western blots represent the relative quantification of each detected band. One representative experiment out of three is illustrated here

In the light of our results, we wondered what the consequences of mitochondrial fragmentation on cell physiology are. As mitochondria are the main source of energy for the cell, we first looked at the ATP production. A 30% decrease in ATP quantity was observed in myoferlin-depleted cells while no modification in ADP or AMP quantity was noticed (Fig. 7a). We then assessed the mitochondrial succinate-tetrazolium dehydrogenase activity (Krebs cycle and respiratory complex II) as an evidence of mitochondrial function in lipogenic Panc-1 and HPAF-2 cell lines depleted for myoferlin. Our results indicated a significant decrease in the activity in both cell lines (Fig. 7b), thus confirming a decrease of the respiratory potential of the cells. Mitochondrial fission may be a preliminary and necessary step for the proper segregation of the organelles between daughter cells during mitosis [30], but may also be a trigger for apoptosis [31] or mitophagy [32]. In our hands, neither the proportion of dying cells (Fig. 7c) nor the caspase-3 and caspase-9 cleavage (Supplemental Figure S6) were increased after myoferlin silencing, while we observed a significant reduction of cell growth (Fig. 7d). Together, these results indicate that myoferlin silencing-mediated mitochondrial fission does not drive cells to mitosis or apoptosis. Damaged mitochondria are considered a source of reactive oxygen species (ROS) for the cells. In our experimental settings, only one siRNA against myoferlin was able to induce a significant ROS production (Fig. 7e). We next reasoned that the lack of energy and the presence of damaged mitochondria in the cells should induce autophagy. Thus, we investigated LC3-II and p62 abundance, the two main autophagy markers. Myoferlin depletion led to a drastic increase in the abundance of these proteins, suggesting an autophagosome accumulation (Fig. 8a).

LC3-II puncta visualization (Fig. 8b) and quantification (Supplemental Figure S7) in immunofluorescence confirmed autophagosome accumulation. Considering that this accumulation can arise from an autophagy induction or from an autophagy late degradation step inhibition, we performed autophagy flux analyses in the presence of several inhibitors: bafilomycin A1 (inhibitor of V-ATPase), chloroquine, or NH_4_Cl (inhibitor of endosomal acidification). Our results demonstrated an additive effect of myoferlin silencing and autophagy inhibitors indicating an autophagy induction by myoferlin depletion (Fig. 8c).

## Discussion

Most of the cancer cells prefer glycolysis to OXPHOS to grow, but successful growth requests a metabolic flexibility. The discovery that resistance to treatment rely on OXPHOS in several cancer types [33–35], including PDAC [9, 13], drew our attention to the importance of identifying the elements rewiring metabolism towards OXPHOS. This is particularly pertinent for PDAC since it is one of the deadliest malignancies with a disappointing increase in overall survival under standard treatment [2, 36].

The present study highlights the importance of myoferlin expression in the description of the survival of patients with early-stage PDAC (mainly stage IIb). Moreover, the abundance of this protein in early-stage (stages IIa and IIb) lesions is significantly and inversely correlated with several ^18^F-DG-PET indices expressing the glycolytic activity: TLG, SUVmean, and SUVmax. In our hands, probably owing to the size of the available cohort, these parameters were not significant survival predictors, unlike what was shown previously in early or advanced PDAC [37, 38].

To our knowledge, the current study demonstrates for the first time that myoferlin silencing deregulates energetic metabolism in lipogenic cell lines, switching them to a glycolytic phenotype. The lipogenic cell subcategory was associated to the PDAC classical subtype described by Collisson et al. [24], while the glycolytic cell subcategory was compared to the PDAC quasi-mesenchymal subtype. Lipogenic cell lines were described as oxidative and used glucose to synthesize lipids through their higher fatty acid synthase activity [11].

It has been known for decades that substrate availability and energetic state modulate the mitochondrial network [25]. Moreover, mitochondrial shaping proteins seem to affect energy production establishing a connection between bioenergetics and fusion/fission machinery [39, 40]. Thus, it appears that mitochondrial morphology is crucially linked to energy metabolism [41]. Enhanced OXPHOS correlates with an interconnected network, whereas low OXPHOS activity and high glycolysis correlates with smaller isolated mitochondria. The ability of neoplastic cells to perform OXPHOS is increasingly seen as a critical point to tumor progression. For example, a recent study showed that circulating breast cancer cells exhibit enhanced mitochondrial biogenesis and their oxidative metabolism to increase their metastatic potential [42].

The role of autophagy in PDAC progression is still controversial [43–45]. However, this catabolic process was demonstrated as a rescue mechanism in PANC-1 cell line under extreme nutrient deprivation conditions [46]. In our study, autophagy failed at restoring proliferation at a level similar to the one observed in control cells. Moreover, we have recently shown that myoferlin silencing led to a reduced pancreatic tumor growth [20].

The metabolic phenotype switch generated by myoferlin silencing could open up a new perspective in the development of combined therapy. To address this specific point, future studies aiming at determining the best chemotherapeutic agent combination are required.

## Materials and methods

### Cells and chemicals

Human PDAC cells BxPC-3 (ATCC CRL-1687), PANC-1 cells (ATCC CRL-1469), and MiaPaCa-2 cells (ATCC CRL-1420) were a generous gift from, respectively, Prof. Bikfalvi (Inserm U1029, Bordeaux, France), Prof. Muller and Burtea (NMR Laboratory, University of Mons, Belgium), and Prof. De Wever (Laboratory of Experimental Cancer Research, University of Gent, Belgium). Cells were authenticated by STR profiling (DSMZ, Braunschweig, Germany). HPAF-2 cells (ATCC CRL-1997) were purchased from ATCC. PaTu8988T [47] (DSMZ ACC162, Braunschweig, Germany) were purchased form DSMZ. All reagents were purchased from Sigma (Bornem, Belgium), unless mentioned otherwise. Antibodies were purchased from Sigma Life Sciences (Bornem, Belgium): myoferlin (HPA014245), vimentin (V6389); Santa Cruz Biotechnology (Santa Cruz, CA): HSC70 (sc-7298), DRP-1 (sc-271583), MIC60/mitofilin (sc-390707), mitofusin (sc-50330); BD Transduction Laboratories: E-cadherin (610181); Cell Signaling (Danvers, MA, USA): phospho-DRP-1 (Ser616) (#4494), p62 (#5114), caspase-3 (#9662), caspase-9 (#9504); Abcam: GAPDH (ab8245), Merck-Millipore (Darmstadt, Germany): mitochondria (MAB1273) or Nanotools (Teningen, Germany): LC3B-2 (0260-100/LC3-2G6).

### Cell culture

PANC-1 cells were maintained in Dulbecco's modified Eagle's medium (DMEM) supplemented with fetal bovine serum (10% fetal bovine serum (FBS)). HPAF-2 cells were cultured in minimum essential medium (MEM) supplemented with FBS (10%), l-glutamine (2 mM), sodium pyruvate (1 mM), and non-essential amino acids for MEM (Gibco, #11140–085). Miapaca-2 cells were cultured in DMEM supplemented with FBS (10%), l-glutamine (4 mM), and sodium pyruvate (1 mM). PaTu8988T cells were maintained in DMEM supplemented with FBS (5%), horse serum (5%), and l-glutamine (2 mM). BxPC-3 cells were cultured in RPMI1640 medium supplemented with 2.5 g/L glucose, 1 mM sodium pyruvate, and 10% FBS. Cells were cultured in a 37 °C, 5% CO_2_ incubator. Cells were checked monthly for mycoplasma and used between passage 1 and 10.

### siRNA transfection

Cells were transfected with 40 nM siRNA targeting myoferlin (Eurogentec, Seraing, Belgium—siRNA#1 CCCUGUCUGGAAUGAGAUUTT; siRNA#2 GAUUGAGGGCCGACAGUUATT), DRP-1 (Dharmacon, Lafayette, CO, USA—Smartpool GAAAGAAGCAGCUGAUAUG, GGAGCCAGCUAGAUAUUA, CAAAGGCAGUAAUGCAUUU, CGUAAAAGGUUGCCUGUUA), DRP-1 5′-UTR (Eurogentec, Seraing, Belgium—GAAACAACGGAAAGAGAAA), or luciferase (Eurogentec, Seraing, Belgium—CUUACGCUGAGUACUUCGATT) as irrelevant siRNA. HPAF-2 cells were transfected during 6 h using Lipofectamine (Life Technologies, Carlsbad, NM, USA) according to the manufacturer’s recommendation. MiaPaCa-2 and PANC-1 cells were transfected with siRNA using calcium phosphate as described previously [48]. All experiments were performed 48 h after transfection, unless mentioned otherwise.

### Plasmid transfection

DRP-1 (NM_005690) expressing plasmid under the control of EF1A promoter was obtained from VectorBuilder (Neu-Isenburg, Germany). Control plasmid was expressing GFP. Plasmids (1 µg) were transiently transfected in Panc-1 cells using Lipofectamine (Life Technologies, Carlsbad, NM, USA) according to the manufacturer’s recommendation. Overnight after plasmid transfection, cells were transfected with siRNA.

### Western blotting

Cells were lysed in sodium dodecyl sulfate (SDS) 1% in the presence of protease and phosphatase inhibitors. SDS-polyacrylamide gel electrophoresis (PAGE) were performed as described previously [23]. Relevance of loading control was evaluated (Supplemental Figure S8).

### Extracellular flux analysis

All experiments were performed with a Seahorse XFp extracellular flux analyzer (Agilent). Cells were seeded (10,000 to 25,000 cells per well) in XFp mini-plates (Agilent) and allowed to attach overnight. For mitochondrial OCR (pmol/min) analysis, cells were kept in unbuffered serum-free DMEM (Basal DMEM, Agilent) supplemented with pyruvate (1 mM), glutamine (2 mM), glucose (10 mM), pH 7.4 at 37 °C, and ambient CO_2_ for 1 h before the assay. During the assay, cells were successively stressed with oligomycin (1 µM), FCCP (1.0 µM, except 0.5 µM for MiaPaCa-2), and rotenone/antimycin A (0.5 µM each) mix. For glycolytic ECAR (mpH/min) measurement, cells were maintained in unbuffered serum-free DMEM (Basal DMEM, Agilent) containing glutamine (2 mM), pH 7.4 at 37 °C, and ambient CO_2_ for 1 h before the assay. During the assay, cells were first added with glucose (10 mM) and then successively stressed with oligomycin (1 µM), and 2-deoxyglucose (50 mM). All results were normalized according to the cell number evaluated by Hoechst (2 µg/mL) incorporation after cold methanol/acetone fixation. Results shown are representative ones out of three independent experiments.

### Mitochondrial TMRE staining

TMRE was used to stain mitochondria in living cells. Transfected cells were seeded in 8-well slides (#80826, IBIDI, Munich, Germany) at low confluence min at 37 °C with TMRE (1 nM) in their respective culture medium. Images were acquired by epifluorescence microscopy as Z-stacks with a Nikon TiS or A1R microscope equipped with a ×100 Oil objective. Results shown are representative ones out of three experiments.

### Immunofluorescence

After siRNA transfection, 5 × 10^4^ cells were seeded on sterile glass coverslips. Forty-eight hours after transfection, cells were washed with Dulbecco's phosphate-buffered saline, fixed with cold methanol/acetone (4:1), and then blocked with 2% bovine serum albumin (BSA) for 30 min. Coverslips were incubated overnight at 4 °C with the primary antibody against phospho-DRP-1 (ser616), or mitochondria diluted in PBS-BSA solution. Coverslips were then washed three times in PBS-BSA solution and incubated with Alexa Fluor 488-conjugated or Alexa Fluor 633-conjugated secondary antibody for 1 h at room temperature. Sections were mounted following three additional washes and nuclei staining with Hoechst (10 ng/mL). Z-stack images were acquired using a Nikon TiS microscope equipped with a high-resolution CCD camera (Andor), and a Nikon ×100 Oil objective. Results shown are representative ones out of three experiments.

### Ultrastructural analysis

Panc-1 cells were fixed for 90 min at room temperature with glutaraldehyde (2.5%) in a Sörensen phosphate buffer (0.1 M, pH 7.4) and post-fixed for 30 min with 2% osmium tetroxide. Embedding and observation were performed as previously described [20].

### ROS measurement

ROS production was measured using CM-H2DCFDA (Invitrogen) fluorescent probe according to the manufacturer’s protocol. Briefly, trypsinized cells were incubated with the probe (2 µM) for 15 min in the dark. After centrifugation, cells were incubated in culture medium during 15 min at 37 °C before fluorescence-activated cell sorting analysis. Results shown are cumulative ones from three independent experiments.

### ATP concentration

The absolute amounts of cellular ATP, ADP, and AMP content were measured using an high-performance liquid chromatography method previously published [49]. Results were expressed as nmol per 10^6^ cells. Results shown are cumulative ones from three independent experiments.

### Succinate dehydrogenase activity

Forty-eight hours after transfection, 8 × 10^3^ transfected cells were seeded in a 96-well plate and allowed to attach overnight. WST-1 reagent (Roche, Mannheim, Germany) was diluted in the culture medium, according to the manufacturer’s instructions. Absorbance at 450 and 620 nm was then measured continuously during 90 min at 37 °C using a Spectramax plate reader (Molecular Devices, Sunnyvale, CA, USA). Slope of the linear portion of the curve was used as succinate dehydrogenase activity. All results were normalized according to the cell number evaluated by Hoechst incorporation. Results shown are cumulative ones from three independent experiments.

### Annexin V/propidium iodide staining

Percentage of apoptotic cells was assessed by FITC-annexin V and propidium iodide staining (BD Biosciences, Franklin Lakes, NJ, USA) according to the manufacturer’s instructions. Flow cytometry data were acquired on a FACSCalibur II™ and data were analyzed using CellQuest™ software (BD Biosciences, Franklin Lakes, NJ, USA). Results shown are cumulative ones from three independent experiments.

### Cell growth

Equal number of cells were seeded in complete medium after transfection and harvested after 48 h. The cell numbers were indirectly determined using Hoechst incorporation. Results were expressed as DNA content. Results shown are representative ones out of three experiments.

### PET/CT, immunochemistry, and assessment

All the ^18^F-DG-PET/CT were performed on two cross-calibrated 3D combined PET/CT scanners (Philips Medical Systems, Cleveland, OH, USA), using classical acquisition and reconstruction protocols, about 65 min after the injection of 3 MBq/kg of ^18^F-DG. Patients fasted at least 6 h before the injection. In the 40 patients who underwent PET/CT, conventional PET parameters were measured on the pancreas lesion including standardized uptake values (SUVmax and SUVmean), MTV, and TLG. TLG was calculated according to the formula: TLG = SUVmean × MTV. For MTV40 and TLG40, segmentation was applied using a threshold method including only the voxels with an SUV ≥40% of the SUVmax of the tumor. Corresponding primary tumors were obtained from our institution Biobank, as formalin-fixed, paraffin-embedded tissue blocks. Sections were stained with antibodies against myoferlin. Sections were then reviewed and scored blindly by three independent investigators (GR, MH, and OP) before access to the positron emission tomography (PET) parameters. Myoferlin scoring was performed by evaluating the intensity (ranging from 0 to 3) and of the extent (%) of each immunolabelled sample. A global score was calculated by the sum of intensities pondered by its respective extent. The maximal intensity was recorded for each tumor as the myoferlin maximal score.

### Ethics statement

All human sections were used with the agreement of the Human Ethic Committee of the University of Liège and from the University Hospital (approval #2016–270). According to Belgian law, patients obtained the information that the residual surgical material could be used for research purpose and the consent is presumed as long as the patient does not oppose.

### Statistics

Kaplan–Meier survival curves were established based on TCGA-PAAD data imported using RTCGA packages [50]. Survival curves were compared using the log-rank test. All other results were reported as means with standard deviation. Two-sided statistical analysis were performed using one-way or two-way analysis of variance depending on the number of grouping factors. Unless mentioned otherwise, group means were compared by unpaired Student’s *t* test or Bonferroni’s post-test according to the group number. Welsch’s correction was applied when homoscedasticity was suspected. Spearman’s rank correlation coefficients were calculated between PET data and myoferlin scoring. *P* < 0.05 was considered as statistically significant. All experiments were performed as several independent biological replicates. Statistics were performed using R v3.4 [51].

## Electronic supplementary material

Figure S1

Figure S2

Figure S3

Figure S4

Figure S5

Figure S6

Figure S7

Figure S8

Table S1

Supplemental Figures
