# Supplementary figures and images for: Myoferlin controls mitochondrial structure and activity in pancreatic ductal adenocarcinoma, and affects tumor aggressiveness
Source: Oncogene. 2018 May 3;37(32):4398–412. doi: 10.1038/s41388-018-0287-z (PMC6085282; doi:10.1038/s41388-018-0287-z)

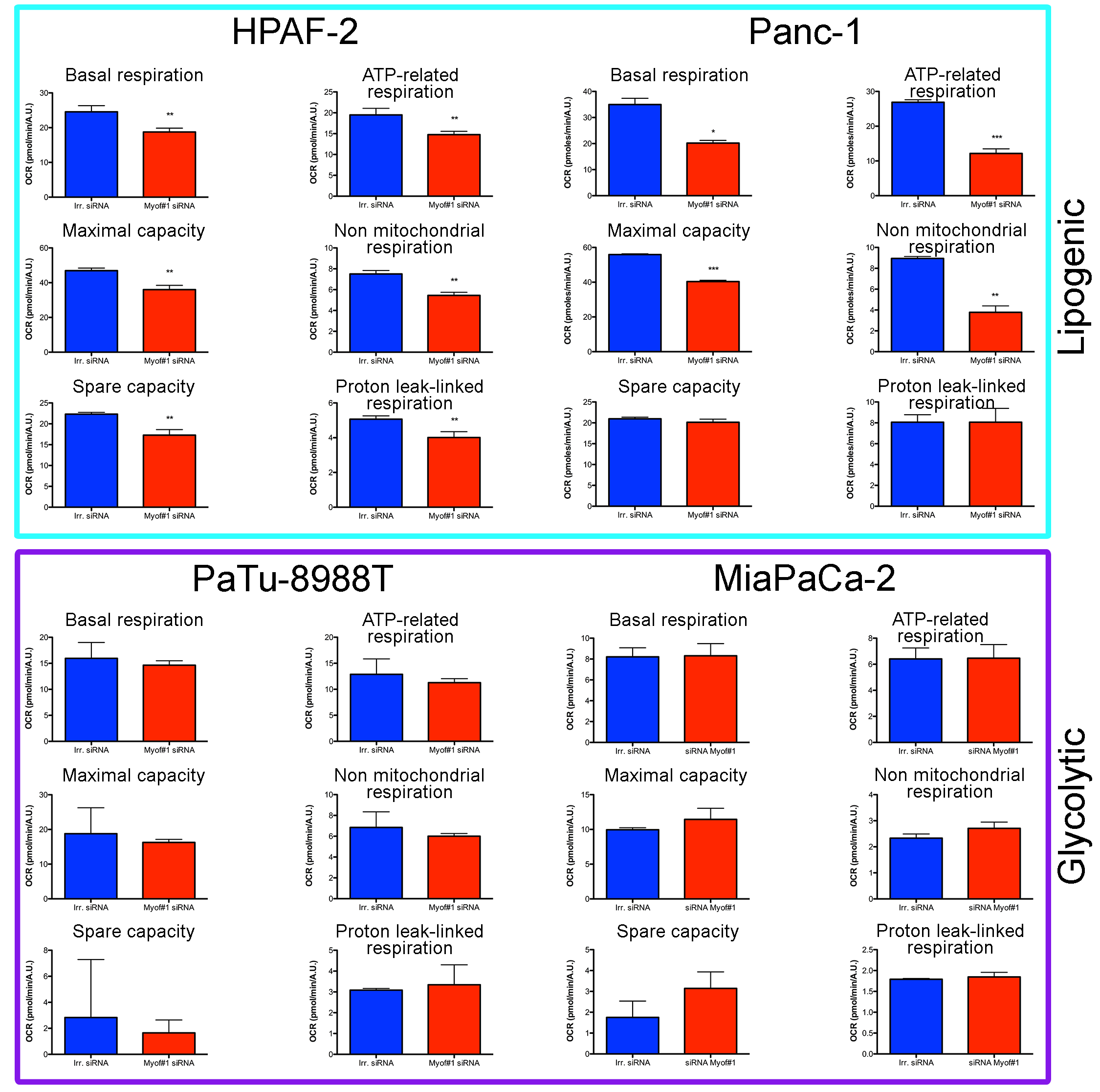

Supplement: Supplementary file 1 — Figure S1 [file 41388_2018_287_MOESM1_ESM.tif]

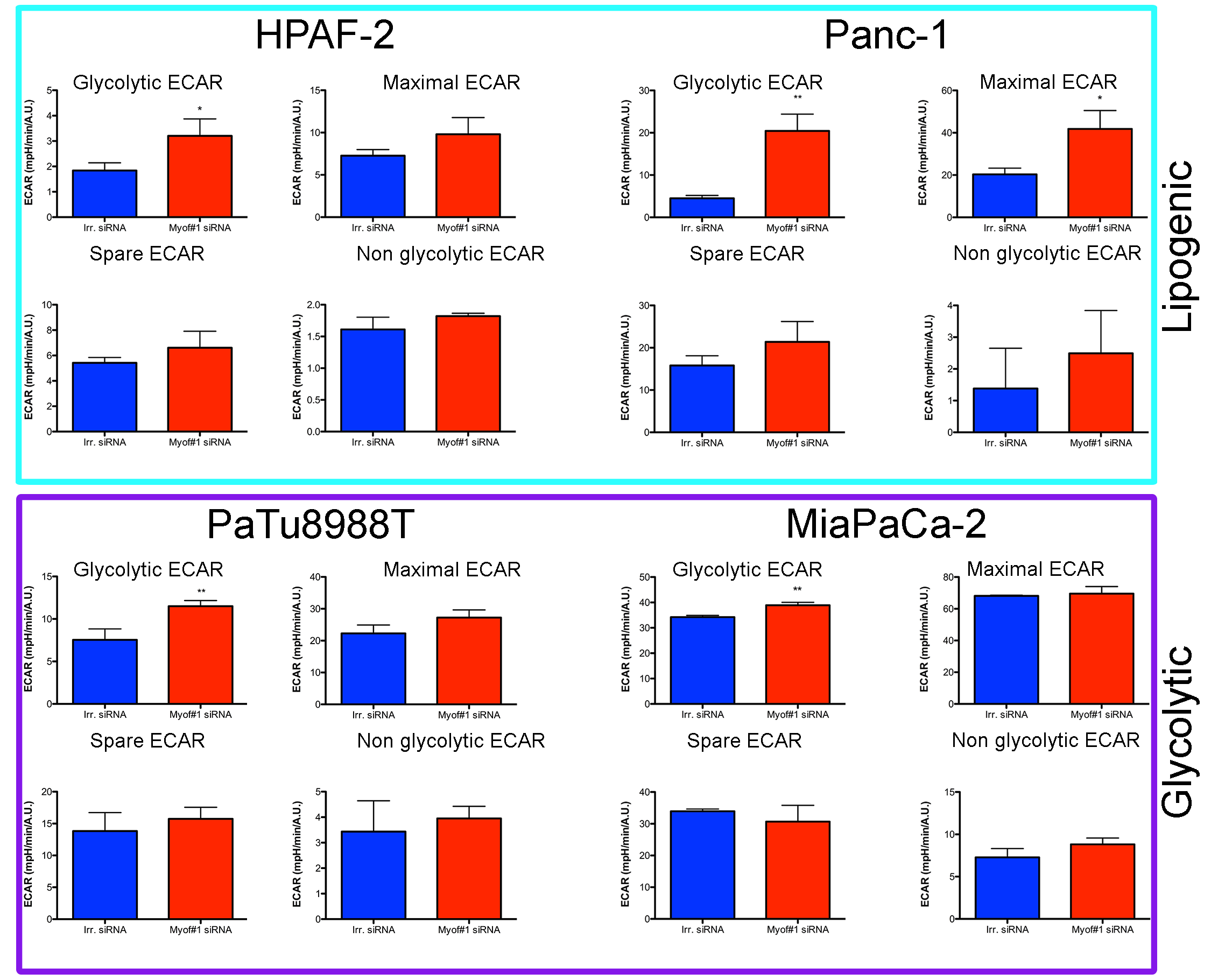

Supplement: Supplementary file 2 — Figure S2 [file 41388_2018_287_MOESM2_ESM.tif]

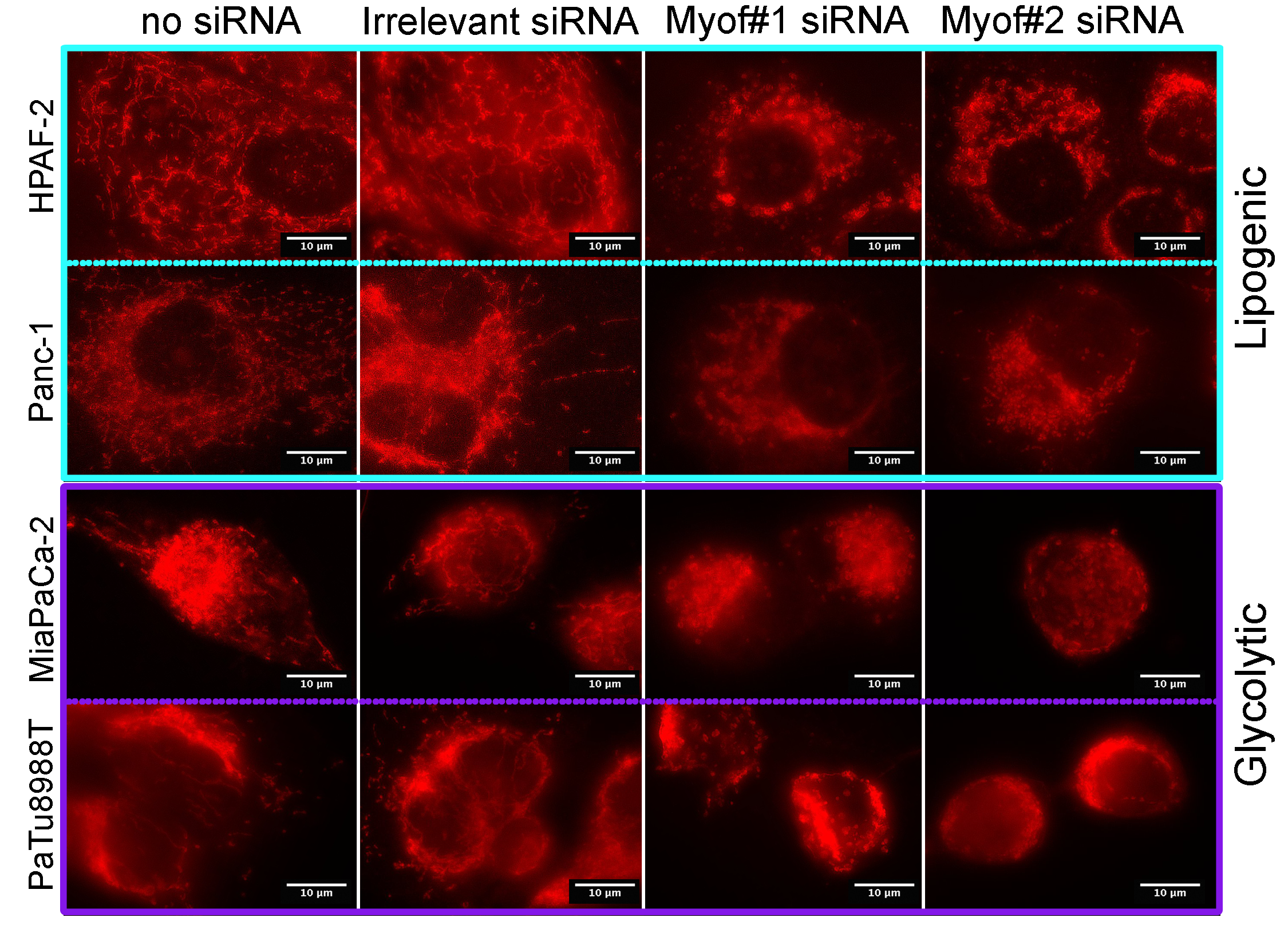

Supplement: Supplementary file 3 — Figure S3 [file 41388_2018_287_MOESM3_ESM.tif]

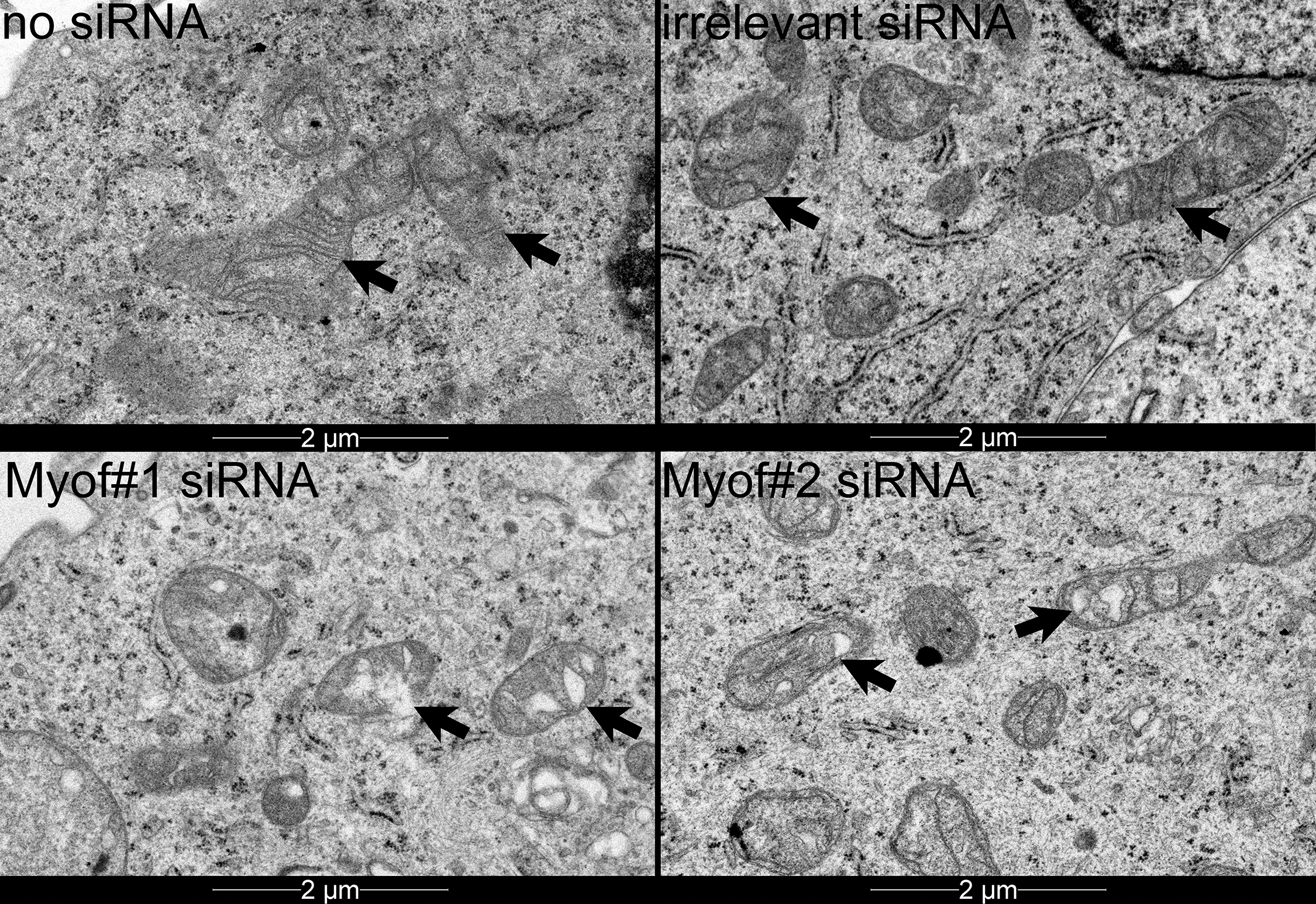

Supplement: Supplementary file 4 — Figure S4 [file 41388_2018_287_MOESM4_ESM.tif]

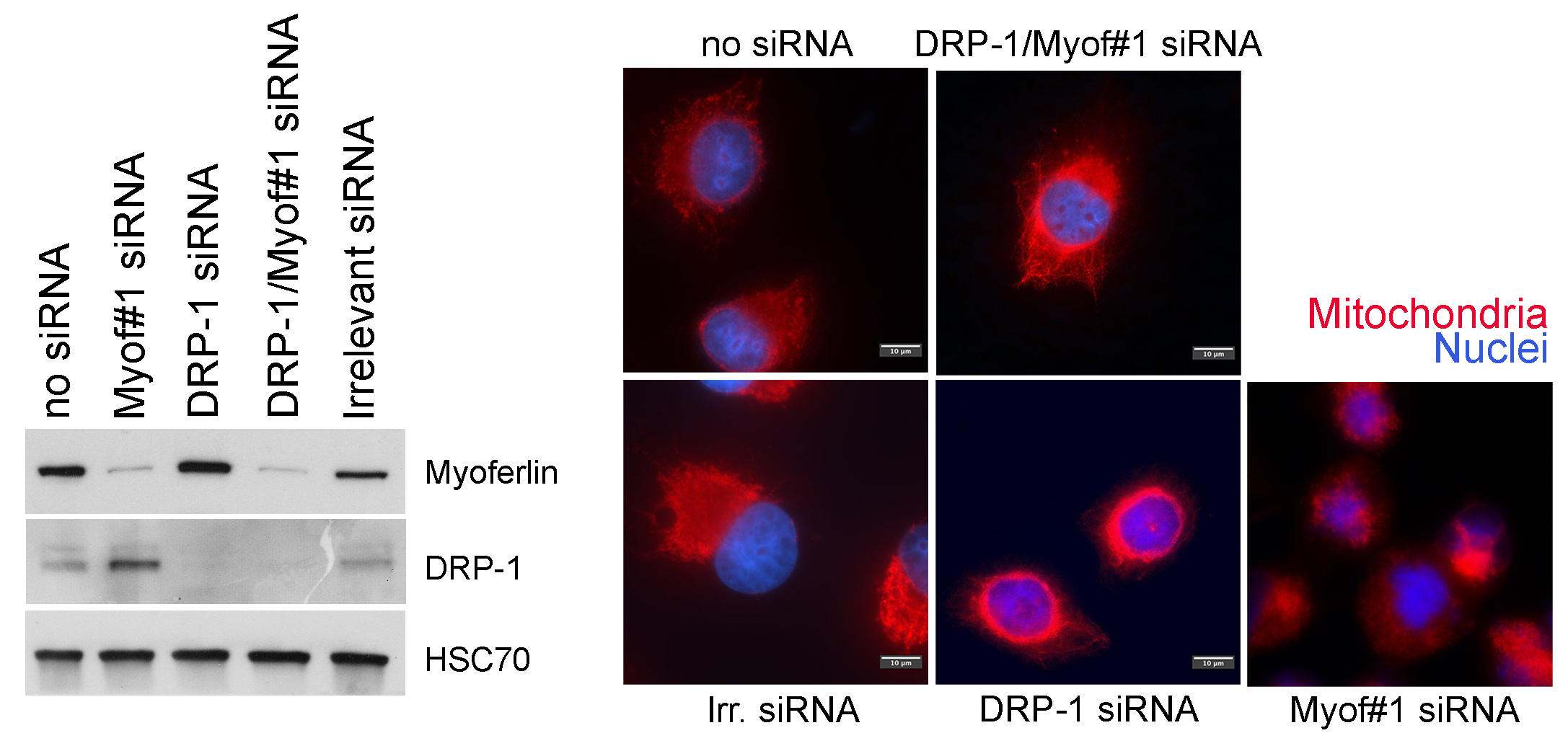

Supplement: Supplementary file 5 — Figure S5 [file 41388_2018_287_MOESM5_ESM.tif]

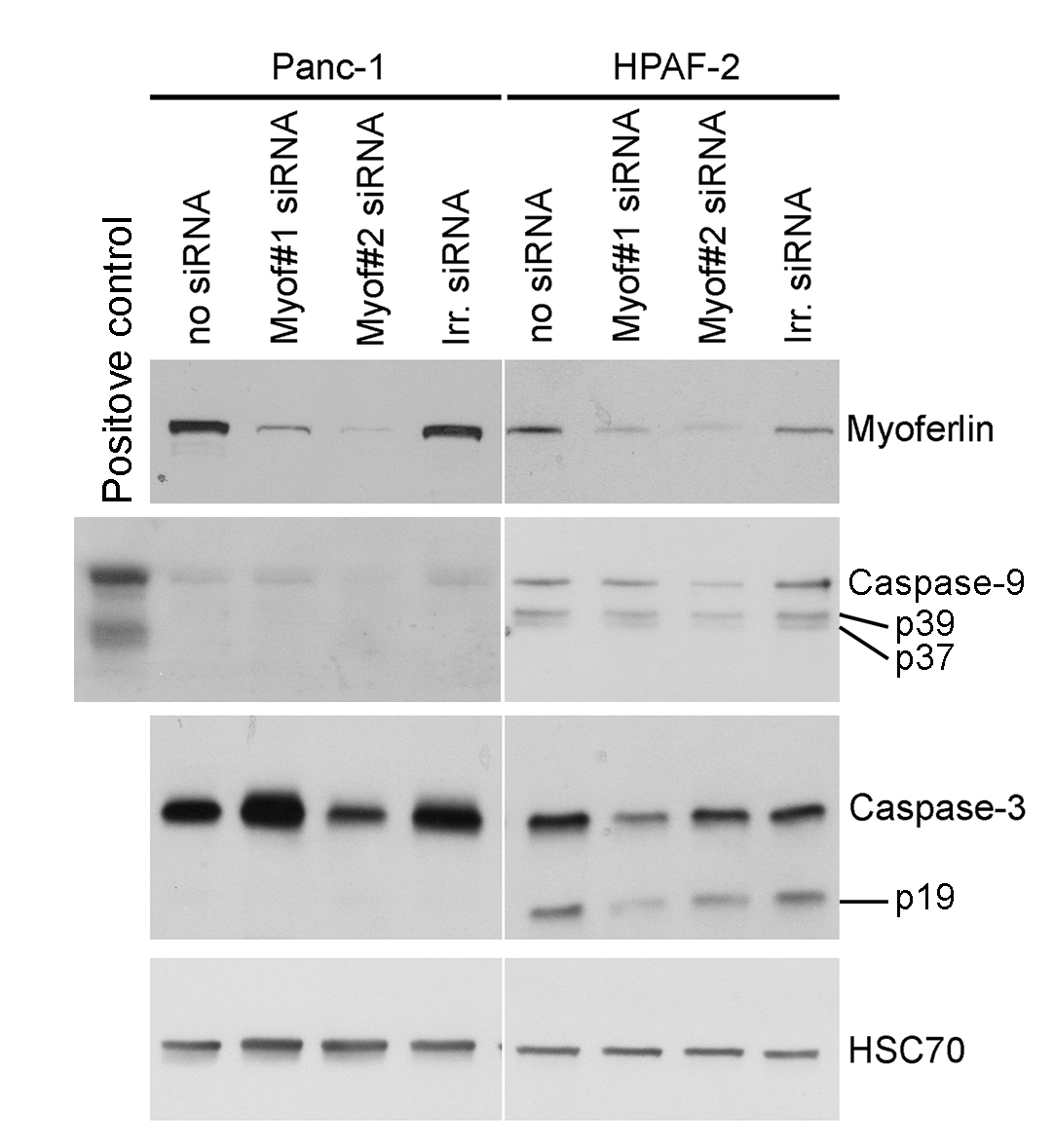

Supplement: Supplementary file 6 — Figure S6 [file 41388_2018_287_MOESM6_ESM.tif]

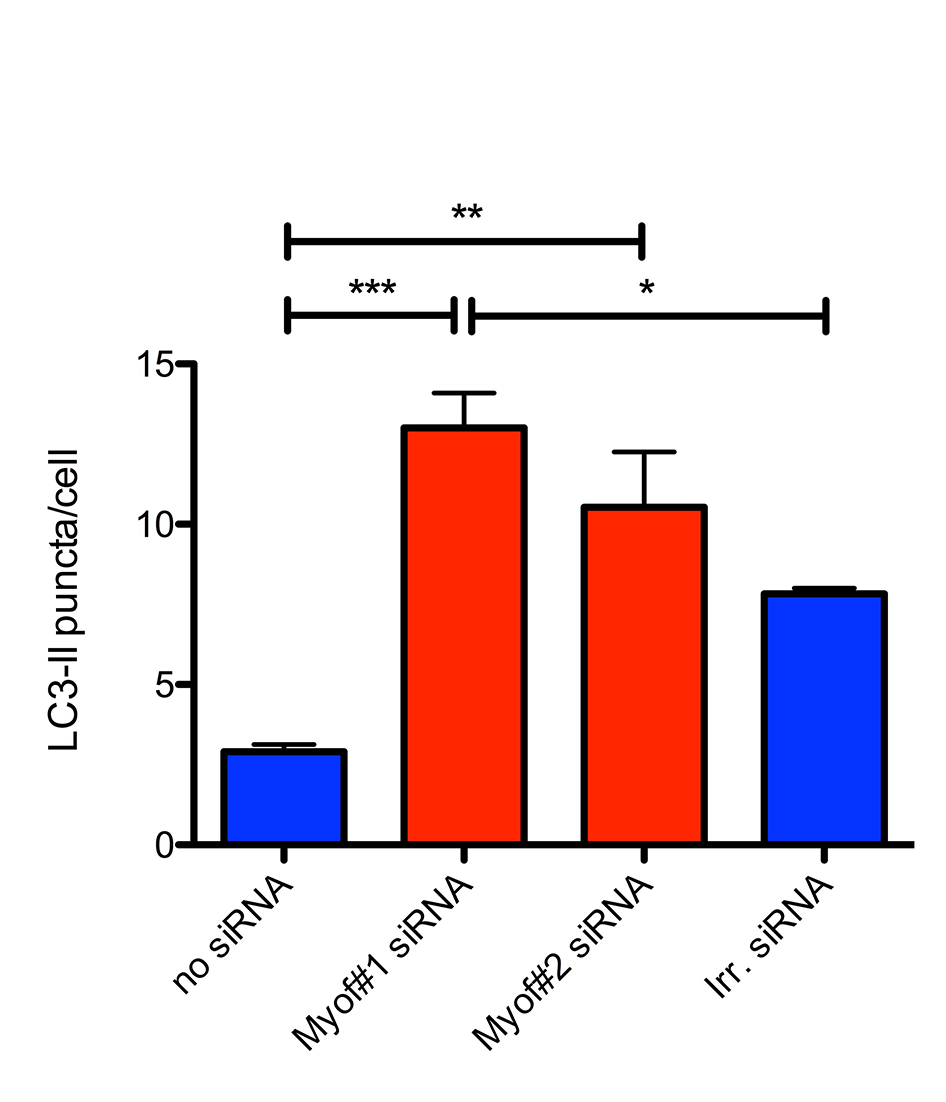

Supplement: Supplementary file 7 — Figure S7 [file 41388_2018_287_MOESM7_ESM.tif]

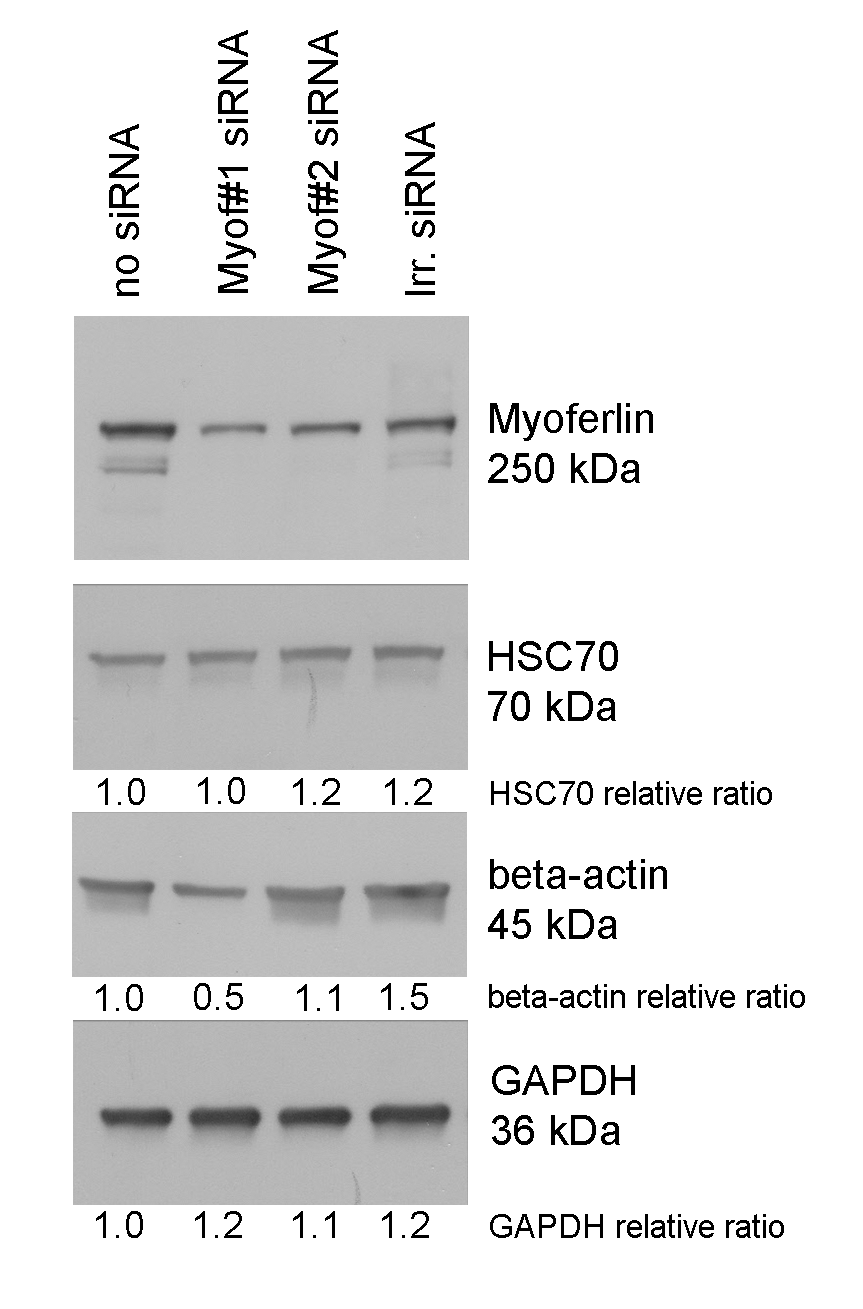

Supplement: Supplementary file 8 — Figure S8 [file 41388_2018_287_MOESM8_ESM.tif]
