# Supplementary material for: Myoferlin controls mitochondrial structure and activity in pancreatic ductal adenocarcinoma, and affects tumor aggressiveness
Source: Oncogene. 2018 May 3;37(32):4398–412. doi: 10.1038/s41388-018-0287-z (PMC6085282; doi:10.1038/s41388-018-0287-z)
Supplement: Supplementary file 9 — Table S1 [file 41388_2018_287_MOESM9_ESM.docx]

Table 1. Description of the PDAC cohort used in myoferlin/PET correlation study

**Gender n (%)**

Female 19 (47.5%)

Male 21 (52.5%)

**Lesion characteristic**

***Localization n (%)***

Head 23 (57.5%)

Body 7 (17.5%)

Tail 10 (25%)

***Stage n (%)***

IIA 7 (17.5%)

IIB 27 (67.5%)

III 2 (5%)

IV 4 (10%)

***Size mean ± s.d.***

3.652 ± 1.286

***PET data mean ± s.d.***

SUVmax 4.892 ± 1.602

SUVmean 2.874 ± 0.961

SUVpeak 4.046 ± 1.413

MTV40 17.54 ± 13.07

TLG40 52.49 ± 52.22
