# Supplementary material for: Myoferlin controls mitochondrial structure and activity in pancreatic ductal adenocarcinoma, and affects tumor aggressiveness
Source: Oncogene. 2018 May 3;37(32):4398–412. doi: 10.1038/s41388-018-0287-z (PMC6085282; doi:10.1038/s41388-018-0287-z)
Supplement: Supplementary file 10 — Supplemental Figures [file 41388_2018_287_MOESM10_ESM.docx]

**Supplemental Figures**

**Figure S1. Oxygen consumption rate (OCR) in PDAC cell lines after myoferlin depletion.**

Basal respiration, ATP-related respiration, maximal respiratory capacity, non mitochondrial respiration, spare capacity, and proton leak-linked respiration of HPAF-2, Panc-1, PaTu8988T and MiaPaCa-2 cells. Each data point represents mean ± SD, n = 3. ****P*<0.001, ***P*<0.01, **P*<0.05.

**Figure S2. Extracellular acidification rate (ECAR) in PDAC cell lines after myoferlin silencing.**

Glycolytic ECAR, maximal glycolytic capacity, spare capacity, and non-glycolytic ECAR of HPAF-2, Panc-1, PaTu8988T and MiaPaCa-2 cells. Each data point represents mean ± SD, n = 3. ***P*<0.01, **P*<0.05.

**Figure S3. Mitochondrial network visualization after myoferlin silencing.**

Immunofluorescence of a mitochondrial 60 kDa non glycosylated protein in Panc-1 cells 48h after myoferlin silencing. Representative experiment out of 3.

**Figure S4. Ultrastructural observation of Panc-1 mitochondria after myoferlin silencing.**

Panc-1 cells depleted for myoferlin during 48h were fixed with glutaraldehyde and observed under transmission electron microscope. Black arrows show representative mitochondria.

**Figure S5. Immunofluorescence observation of Panc-1 mitochondria after myoferlin/DRP-1 silencing.**

Immunofluorescence of a mitochondrial 60 kDa non glycosylated protein in Panc-1 cells 48h after myoferlin and/or DRP-1 silencing. Total protein extract (10 µg) were subjected to SDS-PAGE followed by western blot analysis with specific antibodies against myoferlin or DRP-1.

**Figure S6. Effects of myoferlin silencing on apoptosis.**

Total protein extract (10 µg) were subjected to SDS-PAGE followed by western blot analysis with specific antibodies against myoferlin and (cleaved)-caspase-3 and -9. HSC70 was used as a loading control.

**Figure S7. Effects of myoferlin silencing on autophagosome number.**

Quantification of LC3B-II puncta in Panc-1 cells transfected with myoferlin siRNA. Images were analyzed with Fiji software for cell number and LC3B-II puncta after image thresholding and binarisation. Results are represented as a mean ± SD, n = 290. ****P*<0.001, ***P*<0.01, **P*<0.05.

**Figure S8. Comparison of potential loading control after myoferlin silencing.**

As a glycolytic enzyme, GAPDH was suspected to be a non-pertinent loading control for this study. We compared abundance of HSC-70, beta-actin, and GAPDH after siRNA transfection. It appeared that beta-actin was strongly affected by myoferlin silencing while HSC-70 and GAPDH remained unchanged.
